# Hot Corrosion Behavior and Mechanism of Ru-Containing Ni-Based Single-Crystal Superalloy Exposed to Molten Na_2_SO_4_ at 950 °C

**DOI:** 10.3390/nano16191225

**Published:** 2026-09-28

**Authors:** Shijia Guo, Zhihan Wu, Shengyun Yuan, Bohou Zhang, Cheng Zhou, Fangmiao Duan, Guijuan Zhou, Zhigang Li, Lianxu Yu, Yangtao Zhou, Baobing Zhang, Yong Zhang

**Affiliations:** 1 Herbert Gleiter Institute of Nanoscience, School of Materials Science and Engineering, Nanjing University of Science and Technology, Nanjing 210094, China; 2 Liaoning Academy of Materials, Shenyang 110167, China; 3 China United Gas Turbine Technology Co., Ltd., Beijing 100016, China; 4 Metalink Special Alloys Corporation, Nanjing 211135, China; 5 Shenyang National Laboratory for Materials Science, Institute of Metal Research, Chinese Academy of Sciences, Shenyang 110016, China

**Keywords:** nickel-based single crystal superalloy, Ru addition, high temperature corrosion, molten salts

## Abstract

In this study, the hot corrosion behavior of a low-Cr, Ru-containing nickel-based single-crystal (SX) superalloy under exposure to Na_2_SO_4_ corrosive environments at 950 °C for up to 200 h was systematically investigated. The hot corrosion kinetics of the alloy exhibit a typical two-stage characteristic, consisting of an incubation stage and an acceleration stage. Notably, the mass gain rate is reduced after 100 h of hot corrosion. During the initial incubation stage, the molten sulfate reduces the interfacial oxygen partial pressure, thereby facilitating the formation of discontinuous reticular Al_2_O_3_/Cr_2_O_3_ and metastable MoS_2_. As corrosion progresses, the MoS_2_ in the upper region of the oxide layer is consumed, while a relatively continuous MoS_2_ layer is formed as an internal sulfide layer. In addition, as MoS_2_ evolves and sulfur is continuously supplied from the deposited sulfate, abundant sulfur reacts with Ru to form thermodynamically stable RuS_2_. This indicates that Ru participates in high-temperature sulfidation reactions and accompanies the transformation of corrosion products from the metastable MoS_2_ phase to the thermodynamically stable RuS_2_ phase. The continued mass gain with a reduced rate beyond 100 h of hot corrosion suggests that internal sulfides may influence the sulfur diffusion kinetics, although the inward sulfur penetration remains ongoing.

## 1. Introduction

Nickel-based single crystal (SX) superalloys have been widely used in the hot-end components of advanced aero engines due to their excellent high-temperature mechanical properties and resistance to hot corrosion [1,2,3,4]. Driven by the ongoing increase in aero engine inlet temperatures, fourth-generation nickel-based SX superalloys were developed to meet the increasing requirements for creep resistance at high temperatures [5,6,7,8,9,10]. Compared to previously developed nickel-based SX superalloys, such as the widely used second-generation ones, 2–4 wt.% of Ru has been incorporated into fourth-generation nickel-based SX superalloys to enhance high-temperature mechanical properties and microstructural stability [11]. Meanwhile, due to the relatively high Re content, the Cr content has to be reduced to diminish the formation of topologically close-packed (TCP) phases [12]. Numerous studies have demonstrated that these alloys exhibit superior creep resistance and thermal stability. Nonetheless, sulfur and sodium originating from fuel and salt mist reacted to form Na_2_SO_4_ on the surface of hot-end components. These deposits severely impaired the corrosion resistance of the Ni-based superalloys [13,14,15,16]. However, to the best of the authors’ knowledge, the hot corrosion resistance of fourth-generation SX superalloys has not been investigated yet [17,18,19].

The investigations on the hot corrosion behaviors of SX superalloys have focused on the second-generation nickel-based SX superalloys with high Cr contents [20,21,22,23,24,25,26]. The results show that due to the high Cr content (≥12 wt.%), the Cr element plays a vital role in improving the hot corrosion resistance [27,28]. For instance, Chen et al. investigated the influence of Cr content on the high-temperature corrosion behavior of second-generation SX superalloys and demonstrated that the primary role of Cr is to inhibit sulfide formation, rather than to provide oxidation resistance [27]. Chang et al. examined the interaction between Ta and Cr in relation to the Type-I hot corrosion resistance of second-generation SX superalloys [28]. Their findings revealed that a thick sulfate layer forms on the surface of the Cr_2_O_3_ oxide layer, effectively reducing the oxygen partial pressure at the Cr_2_O_3_/sulfate interface. This decrease in oxygen partial pressure subsequently suppresses the dissolution of Cr_2_O_3_, thereby improving the alloy’s resistance to hot corrosion. Therefore, it can be concluded that in the second-generation nickel-based SX superalloys with high Cr content, the primary protective role of chromium is twofold. Under low-oxygen conditions, chromium effectively inhibits sulfur diffusion via the “sulfide blocking effect”; under high-oxygen conditions, it promotes the formation of protective Cr_2_O_3_ oxide layers, thereby obstructing the penetration pathways of corrosive media.

In addition, Lin et al. [29] and Guan et al. [30] investigated the effect of Ru content on the hot corrosion performance of high Cr (≥12 wt.%) nickel-based polycrystalline superalloys. The results indicated that Ru promotes the segregation of Cr and Mo in the dendritic regions, thereby facilitating the formation of continuous and dense Al_2_O_3_ and Cr_2_O_3_ oxide layers. Moreover, Ru also mitigates spallation of corrosion products, reduces the corrosion weight gain rate, decreases the thickness of the corrosion layer, and enhances the adhesion between the corrosion layer and the substrate, ultimately leading to a significant improvement in the hot corrosion resistance of high-Cr nickel-based superalloys. Therefore, it can be concluded that existing studies have shown that in high-Cr alloys, the role of Ru addition is to influence the formation of dense Al_2_O_3_ and Cr_2_O_3_ layers [31]. However, in fourth-generation nickel-based SX superalloys, the Cr content (2–4 wt.%) is significantly lower than that of the as-cast nickel-based alloys (≥12 wt.%) examined in the aforementioned studies. Therefore, one of the primary objectives of this study is to determine whether the reduced Cr content is sufficient to maintain a continuous Cr_2_O_3_ layer comparable to that formed in high-Cr nickel-based superalloys, which typically serves as a protective barrier against inward diffusion of sulfur. Furthermore, according to thermodynamic equilibrium calculations reported in the literature [32], Ru is thermodynamically prone to react with sulfur to form RuS_2_ at 950 °C, and the equilibrium amount of RuS_2_ increases slightly with rising temperature. However, the sulfidation-related behavior of Ru in low-Cr fourth-generation nickel-based single-crystal superalloys under molten Na_2_SO_4_ hot-corrosion conditions has not been systematically verified. Thereby, the second objective of this study is to elucidate the potential role of Ru and the formation mechanism of Ru sulfide in the fourth-generation SX superalloys under hot corrosion conditions.

Therefore, this study systematically investigated the hot corrosion behavior of a low-Cr, Ru-containing fourth-generation nickel-based single-crystal superalloy under exposure to Na_2_SO_4_ corrosive media at 950 °C. The evolution of corrosion kinetics and corrosion products as a function of exposure time was analyzed. These results offer new insights for optimizing the high-temperature corrosion resistance of the fourth-generation nickel-based SX superalloys.

## 2. Experiment

### 2.1. Material

Single-crystal specimens were prepared by the liquid-metal-cooled (LMC) directional solidification method using an LVM-2 furnace (Russian Academy of Sciences, Saint Petersburg, Russia). The nominal composition of the SX superalloys is listed in Table 1 (all data are given in wt.%). The SX superalloy rods were fabricated via directional solidification by the liquid metal cooling (LMC) process with a drawing rate of 3 mm/min. The seed crystal with [001]-orientation was used to ensure that the SX bar grows with the desired orientation. The SX bar was then subjected to the solutionized heat treatment of 1285 °C/2 h, 1300 °C/2 h, and 1325 °C/20 h and a two-step aging treatment at 1100 °C/4 h/air cooling (AC) and 870 °C/24 h/AC. The hot corrosion specimens with a dimension of 14 mm in diameter and 3 mm in height were cut by means of wire electrical discharge machining. The specimen surfaces were ground with #240, #400, #600, #800 and #1200 SiC papers, and then cleaned with acetone and ethanol to remove contamination.

### 2.2. Hot Corrosion Test

Hot corrosion testing was conducted via the sulfate deposition method. The specimen was cleaned in a 3:1 ethanol–acetone solution, dried under ambient conditions, and weighed to determine the initial mass. Subsequently, the samples were placed on a clean nickel plate preheated to 150 °C and uniformly coated with a saturated Na_2_SO_4_ solution using a fine-bristled brush. Sulfate loading was controlled at 0.3–0.5 mg/cm^2^ per corrosion cycle and verified gravimetrically using an analytical balance with an accuracy of 0.01 mg. Following coating, the specimen was transferred into a pre-weighed corundum crucible, which was then subjected to isothermal corrosion in a muffle furnace at the designated temperature of 950 °C. Each exposure cycle had a duration of 25 h. Upon completion of a cycle, the crucible was withdrawn from the furnace, promptly sealed with lids, and air-cooled for 10 min. The specimen was then immersed in boiling deionized water for 20 min to remove residual salts, which is a standard procedure for hot corrosion experiments [33,34,35,36]. No discernible delamination or loss of solid corrosion products (i.e., oxides and sulfides) was observed during the treatment. After thorough drying in ambient air, the specimen-crucible assembly was reweighed to include any spalled corrosion product fragments retained within the crucible. Afterward, the salt layer was reapplied, and the specimen was returned to the original crucible and reintroduced into the furnace for the subsequent cycle. The experimental cycle was repeated iteratively until the total accumulated corrosion exposure time reached 200 h (Figure 1); each data point of the mass change was the average of three samples.

### 2.3. Microstructural Characterizations

The microstructural characterization of the SX superalloy was performed using a FEI Talos F200X transmission electron microscope (TEM, Thermo Fisher Scientific, Hillsboro, OR, USA) equipped with an energy-dispersive X-ray spectroscopy (EDX) detector, operated at 200 kV. TEM specimens (3 mm diameter disks) were cut from the SX cylinder and mechanically ground to a thickness of approximately 50 μm. TEM foils were further thinned via electrochemical polishing using a Struers Tenupol-5 twin-jet electropolisher (Struers, Ballerup, Denmark). The electrolyte consisted of 10 vol.% perchloric acid and 90 vol.% ethanol, with the polishing temperature maintained at −20 °C and an applied voltage of 20 V.

Corrosion products were characterized by X-ray diffraction (XRD, Bruker-AXS D8 Advance, Bruker AXS GmbH, Karlsruhe, Germany) and scanning electron microscopy (SEM, Gemini SEM 360, Carl Zeiss Microscopy GmbH, Jena, Germany) equipped with an EDX detector. Site-specific TEM lamellae of corrosion products were prepared using a focused ion beam (FIB) system (FEI Helios 5UX, Thermo Fisher Scientific, Hillsboro, OR, USA).

## 3. Results

### 3.1. Sx Superalloy Microstructure

Figure 2a shows the HAADF-STEM image of the representative microstructure of the SX superalloys and the electron diffraction pattern along the [001] zone axis. The STEM-EDX elemental maps are shown in Figure 2b–j. Apparently, Al and Ta are partitioned in the γ′ phase, while Co, Cr and Re are primarily enriched in the γ matrix. Note that Ru also shows a slight preference to be in the γ matrix, though the partitioning of Ru between the γ′ phase and γ matrix is not as discernible as that of Co, Cr and Re. Mo and W do not exhibit remarkable partitioning between the γ′ phase and γ matrix.

### 3.2. Hot Corrosion Kinetics

Figure 3 illustrates the hot corrosion kinetic curves of the SX superalloy specimen coated with molten sulfate at 950 °C. After 200 h of hot corrosion exposure at 950 °C, the weight gain per unit area on the specimen surface reaches 90.6 mg/cm^2^. During the initial 0–25 h incubation period, the weight gain rate is low, with the cumulative weight gain at 25 h being less than 4.1 mg/cm^2^. This could be attributed to the uniform coverage of a molten Na_2_SO_4_ salt film on the specimen surface in the early stage of hot corrosion. The Na_2_SO_4_ salt film served as the barrier layer for effectively isolating the substrate from the surrounding atmospheric environment. As a result, the oxygen required for high-temperature oxidation of the alloy was primarily supplied by the molten Na_2_SO_4_ salt, thereby inhibiting the rapid progression of hot corrosion. In the 25–100 h stage, the weight gain rate increases rapidly, suggesting severe corrosion occurred on the specimen surface during this period. When the corrosion time extends to 100–200 h, the weight gain rate is lowered, suggesting that a certain protective oxide layer was formed on the surface of the specimen, which could prevent the corrosion medium from penetrating inward. However, the density of this oxide film was insufficient, and its protective efficiency was limited, unable to effectively prevent the continuous inward diffusion of corrosive components such as oxygen and sulfur.

### 3.3. XRD

Figure 4 presents the XRD spectra of the specimen surface subjected to hot corrosion at 950 °C for various times. After 25 h of corrosion at 950 °C, the corrosion products formed on the surface are composed of NiO, Al_2_O_3_, NiCr_2_O_4_ and MoS_2_. When the thermal corrosion time was extended to 125 h, characteristic diffraction peaks corresponding to NiO and Al_2_O_3_ remained discernible in the XRD spectrum, while the peak of the MoS_2_ sulfide intensified. After 200 h of hot corrosion, NiO and Al_2_O_3_ are predominant corrosion products on the specimen surface, whereas MoS_2_ remains detectable.

### 3.4. Morphologies and Microstructures During Hot Corrosion

Figure 5 presents the representative microphotographs of microstructures and elemental distribution characteristics of the present SX superalloy after hot corrosion at 950 °C for 25 h. After 25 h exposure, EDX mapping results reveal that the outer oxide layer is mainly composed of oxides of Ni, Al, Cr, W, Ru, and Re. Al_2_O_3_ is distributed as a discontinuous network (Point 1 in Table 2). Above the network-like Al_2_O_3_ layer, numerous Ta-bearing oxide particles and Mo-based sulfides are distributed. Within the underlying sulfide layer, the main sulfide product is MoS_2_ (Point 3 in Table 2), and a small amount of Cr sulfide (Point 2 in Table 2) can also be observed.

When the hot corrosion time was extended to 125 h (Figure 6), the thickness of the corrosion layer significantly increased, and its spatial distribution became more uniform. A spinel oxide layer consisting of Ni/Al/Cr/W/Ta/Re mixed oxides was formed between the NiO and discontinuous reticular Al_2_O_3_/Cr_2_O_3_ layers. Compared with the state at 25 h, this spinel layer partially suppressed the inward diffusion of oxygen and sulfur, which might contribute to the lowered mass gain rate. Meanwhile, the amount of Cr sulfide in the sulfide layer increased. The outward-diffused Cr element reacted with the sulfur to form the Cr sulfide, which might inhibit the continuous inward diffusion of the sulfur element to a certain extent [20].

When the duration of hot corrosion was extended to 200 h (Figure 7), the thickness of the corrosion layer further increased, and the microstructure of the oxide layer tended to stabilize. The upper part of the intermediate layer consists of a spinel oxide layer composed of mixed Al/Cr/Co oxides. Beneath this spinel layer lies a relatively continuous oxide layer enriched with W, Ta and Re. Further downwards, the originally network-like Al_2_O_3_ within the internal-oxidation zone transforms into an approximately planar structure. In addition, a relatively continuous MoS_2_ layer is formed underneath the network-like Al_2_O_3_.

To further explore the microstructure of the oxide film, FIB was utilized to lift out the TEM specimens from the oxide layer in the specimen after hot corrosion at 950 °C for 25 h. Figure 8a shows the representative high-angle annular dark-field scanning transmission electron microscope (HAADF-STEM) image of this region and the corresponding EDX elemental map. The white regions are significantly enriched with Ni, Co, Ta, W, Cr, Re, and Ru. The selected area electron diffraction (SAED) pattern (SAED 1 in Figure 8b) indicates that this region is composed of the cubic γ matrix phase. TEM observations confirmed that MoS_2_ particles were formed in the oxide layer, as revealed by the compositional analysis (see Figure 8a and Table 3) and SAED pattern 2 in Figure 8b. In addition, Cr-rich oxide particles are intermittently embedded in the reticular Al-rich oxides.

Figure 9a presents a representative HAADF-STEM image of the sample following 125 h of hot corrosion, along with the corresponding EDX elemental distribution maps. As the corrosion duration increased, the absence of an effective protective oxide layer during the initial stage of hot corrosion resulted in direct exposure of the matrix to the surrounding environment, thereby promoting extensive oxidation of the matrix. As a result, the outermost layer is composed of NiO grains of varying sizes (SAED 1 in Figure 9b). In the intermediate spinel oxide layer, the regions that remained unoxidized regions during the early corrosion stage have been fully oxidized. Al/Cr-rich oxide strips (SAED 3 in Figure 9b) remain sparsely distributed and have not yet developed into a continuous, dense lamellar structure. Meanwhile, sulfur diffused inward and reacted with Ru to form RuS_2_ (SAED 2 in Figure 9b). The SAED pattern of RuS_2_ is shown in the Supplementary Materials (Figure S1). In addition, dispersed W/Ta-rich oxide particles were observed.

## 4. Discussion

### 4.1. Hot Corrosion Process

As shown in Figure 3, the hot corrosion kinetic curve of the Ru-containing Ni-based single-crystal superalloys at 950 °C can be divided into two stages, i.e., the corrosion incubation period and corrosion acceleration period, which is analogous to the characteristic two stages observed in the second-generation SX alloys at 900 °C [28,33,34]. Nonetheless, a reduced mass gain rate was observed after 100 h of hot corrosion in the corrosion kinetic curve. Therefore, the following discussion will focus on the evolution processes and underlying mechanisms of hot corrosion of the present fourth-generation SX alloy at elevated temperatures.

Based on SEM and TEM microstructural characterization, the corrosion products and underlying processes are schematically shown in Figure 10. At the initial hot corrosion stage (Figure 10a), the specimen surface is evenly covered by a continuous molten sulfate layer, which serves as an isolation layer separating the matrix from the surrounding atmospheric environment. The elements Ni, Al, Cr, Ta and W in the matrix diffused to the specimen surface promptly and reacted with the molten salt to form various corrosion products at different depths. As shown in Figure 4, the oxygen participating in interfacial oxidation was primarily derived from the decomposition of molten Na_2_SO_4_, which remarkably reduced the oxygen partial pressure at the salt-matrix interface. Such a low oxygen potential promoted the selective internal oxidation of Al and Cr, generating network-like Al_2_O_3_/Cr_2_O_3_ in the subsurface zone [31,37,38,39], as shown in Equations (1)–(3):

(1) $${Na}_{2}SO_{4}={Na}_{2}O+\frac{1}{2}S+\frac{3}{2}O_{2}$$

(2) $$2Cr+\frac{3}{2}O_{2}={Cr}_{2}O_{3}$$

(3) $$2Al+\frac{3}{2}O_{2}={Al}_{2}O_{3}$$

Meanwhile, the low interfacial oxygen partial pressure enabled the preferential reaction between Mo and sulfur, forming initial MoS_2_ precipitates in the subsurface at the corrosion incubation period. Moreover, sulfur exhibits a higher diffusivity than oxygen in the Ni-based superalloy matrix [28]. Consequently, although the majority of sulfur reacted with Mo in the shallow oxide zone during the initial stage of hot corrosion, a minor fraction continuously penetrates inward. Upon traversing the discontinuous surface oxide scale, sulfur reacted with Cr and Mo in the alloy matrix to form CrS and MoS_2_. The following equations describe these reactions:

(4) $$Mo+2S=MoS_{2}$$

(5) Cr+S=CrS

As exposure time increased in the incubation period, the sulfur penetration depth progressively increased, culminating in the formation of an internal sulfidation zone. This stage corresponds to the corrosion incubation period on the hot corrosion curve (Figure 3).

As hot corrosion proceeded (Figure 10b), continuous growth of the oxide scale gradually consumed the surface molten sulfate, significantly increasing the local activity of oxygen and sulfur at the sulfate–specimen interface. As a result, atmospheric oxygen can directly reach the specimen surface and engage in interfacial reactions. As displayed in Figure 6, a dense and continuous NiO outer layer and a spinel intermediate layer were progressively formed. Numerous MoS_2_ products were retained in the lower region of the oxide layer. The absence of MoS_2_ products from the upper region may be attributed to its oxidation-driven conversion into volatile MoO_3_ [40,41]. KC et al. conducted density functional theory calculations to investigate the interaction of the MoS2 surface with oxygen [42]. It was found out that the adsorption of oxygen and oxidizing MoS2 is thermodynamically favored, as the Mo–O bond is stronger compared to Mo–S. Such oxidation progresses according to the following stoichiometry [40]:

(6) $$2MoS_{2}+7O_{2}=2MoO_{3}+4SO_{2}$$

The locally released sulfur can then diffuse and react with Ru to form RuS_2_ [43].

(7) $$Ru+2S=RuS_{2}$$

After 200 h of hot corrosion (Figure 10c), the overall corrosion scale thickness increases significantly, and the oxide layer becomes more uniform and stable. The MoS_2_ phase in the oxide layer completely disappears (Figure 7). In contrast, a continuous internal sulfidation layer mainly composed of MoS_2_ forms at the interface between the γ′-denuded zone and the alloy matrix. Notably, the formation of a MoS_2_ layer has seldom been reported in earlier investigations on second-generation SX superalloys [40]. In previous work, the high Cr concentration of Cr facilitated the formation of a dense Cr_2_O_3_ layer, which acted as an effective barrier against inward diffusion of sulfur and oxygen [44,45,46]. In contrast, the present SX alloy with a lower Cr content developed a discontinuous reticular Al_2_O_3_/Cr_2_O_3_ layer, which is less effective as a diffusion barrier for oxygen and sulfur compared to the continuous Cr_2_O_3_ scale in the second-generation SX superalloys. The formed MoS_2_ layer may locally sequester sulfur at the corrosion front rather than fully stopping sulfur penetration. As a result, the internal sulfide layer gradually propagates deeper into the alloy substrate with prolonged corrosion time. This accounts for the deceleration in mass gain observed after 100 h of hot corrosion, as displayed in the hot corrosion curve shown in Figure 3. Nonetheless, the reduction in mass-gain rate after 100 h should not be interpreted as a genuine improvement in the hot corrosion resistance. The formation of internal sulfides alters the diffusion kinetics of oxygen and sulfur, leading to a decreased mass-gain rate, while inward sulfur penetration and substrate degradation still persist.

### 4.2. Role of Ru

Ru-containing fourth-generation SX superalloys exhibit distinct corrosion product evolution and microstructural response under molten sulfate-induced corrosion at 950 °C. During prolonged hot corrosion at 950 °C, the oxygen partial pressure at the corrosion interface gradually increases, which might trigger the oxidative decomposition of MoS_2_ formed in the early corrosion stage. As a result, the phase fraction of MoS_2_ continuously decreased, accompanied by the release of free sulfur. The abundant sulfur in the system further reacted with Ru in the matrix to in situ form a new phase of RuS_2_. This observation indicates that in the Ru-containing fourth-generation SX superalloy, Ru participated in high-temperature sulfidation reactions and may accompany the transformation of corrosion products from the metastable MoS_2_ phase to the thermodynamically stable RuS_2_ phase. Although both MoS_2_ and RuS_2_ may locally sequester sulfur at the corrosion front, they could not block the continuous inward migration of sulfur, and the sulfide layer steadily propagated deeper into the alloy substrate with increasing corrosion time. The reduced mass-gain rate after 100 h was not caused by sulfur inhibition, but originated from the transition of dominant corrosion mechanisms.

## 5. Conclusions

The hot corrosion behavior and microstructural evolution of a Ru-containing fourth-generation Ni-based SX superalloy subjected to molten sulfate-induced corrosion at 950 °C were systematically investigated. The main conclusions are drawn as follows:

1. The hot corrosion kinetic process of the present SX superalloy exhibits a characteristic two-stage behavior: an incubation stage followed by an acceleration stage. Notably, however, the mass gain rate decreased after 100 h of exposure to hot corrosion conditions.
2. In the present Cr-lean SX superalloy, the Al_2_O_3_/Cr_2_O_3_ layer exhibited a discontinuous reticulated morphology owing to insufficient Cr, and thus provided only limited resistance to inward oxygen and sulfur transport. This complied with the accelerating corrosion stage after 25 h exposure. With prolonged exposure, the abundant sulfur reacted with Mo to form the MoS_2_ phase. Particularly, a stable internal sulfide layer was eventually formed at the interface between the γ′-denuded zone and the substrate.
3. In the present Ru-bearing fourth-generation nickel-based superalloy, in situ generated RuS_2_ was observed in the corrosion products. This indicated that Ru participated in high-temperature sulfidation reactions and may accompany the transformation of corrosion products from the metastable MoS_2_ phase to the thermodynamically stable RuS_2_ phase.

## Figures and Tables

**Figure 1:**
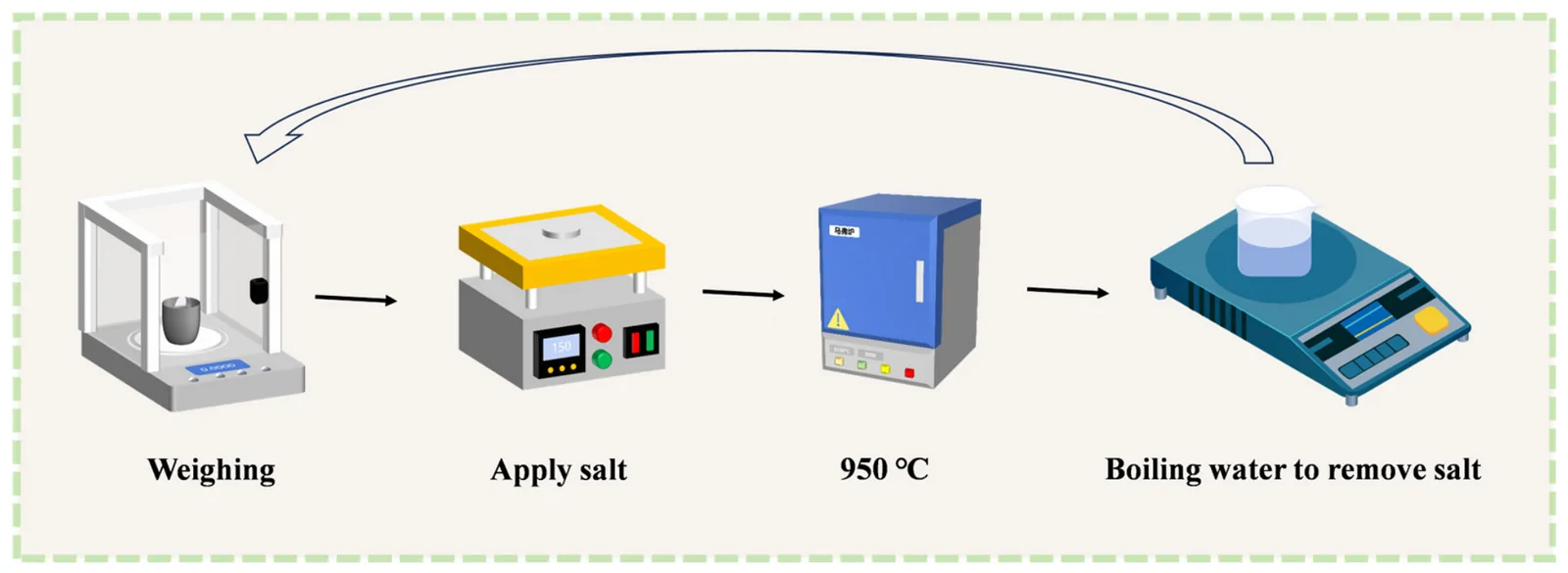
Schematic illustration of the experimental procedure.

**Figure 2:**
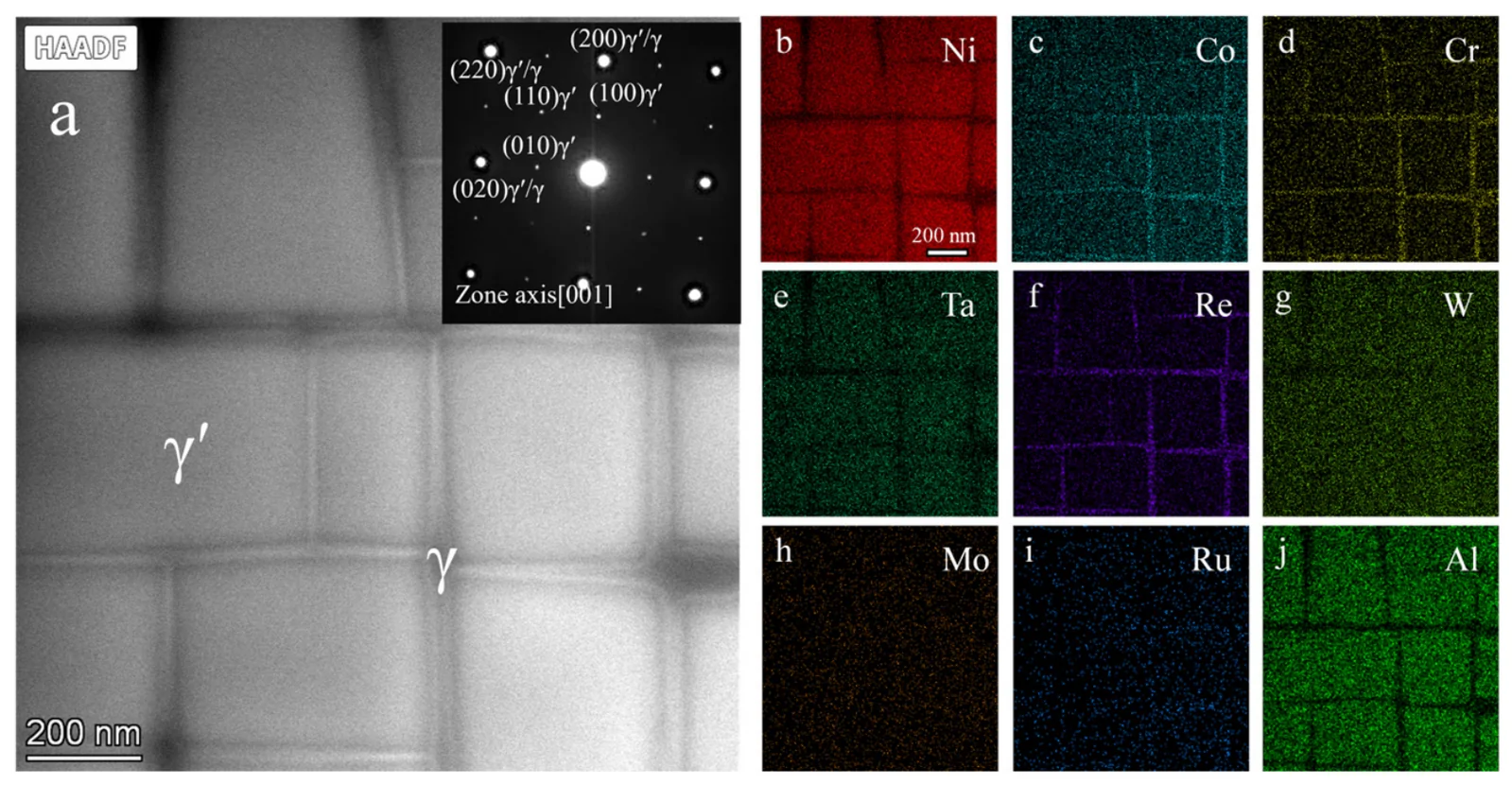
(**a**) The HAADF-STEM image of the representative microstructure of the SX superalloy and the corresponding electron diffraction pattern; (**b**–**j**) STEM-EDX elemental maps.

**Figure 3:**
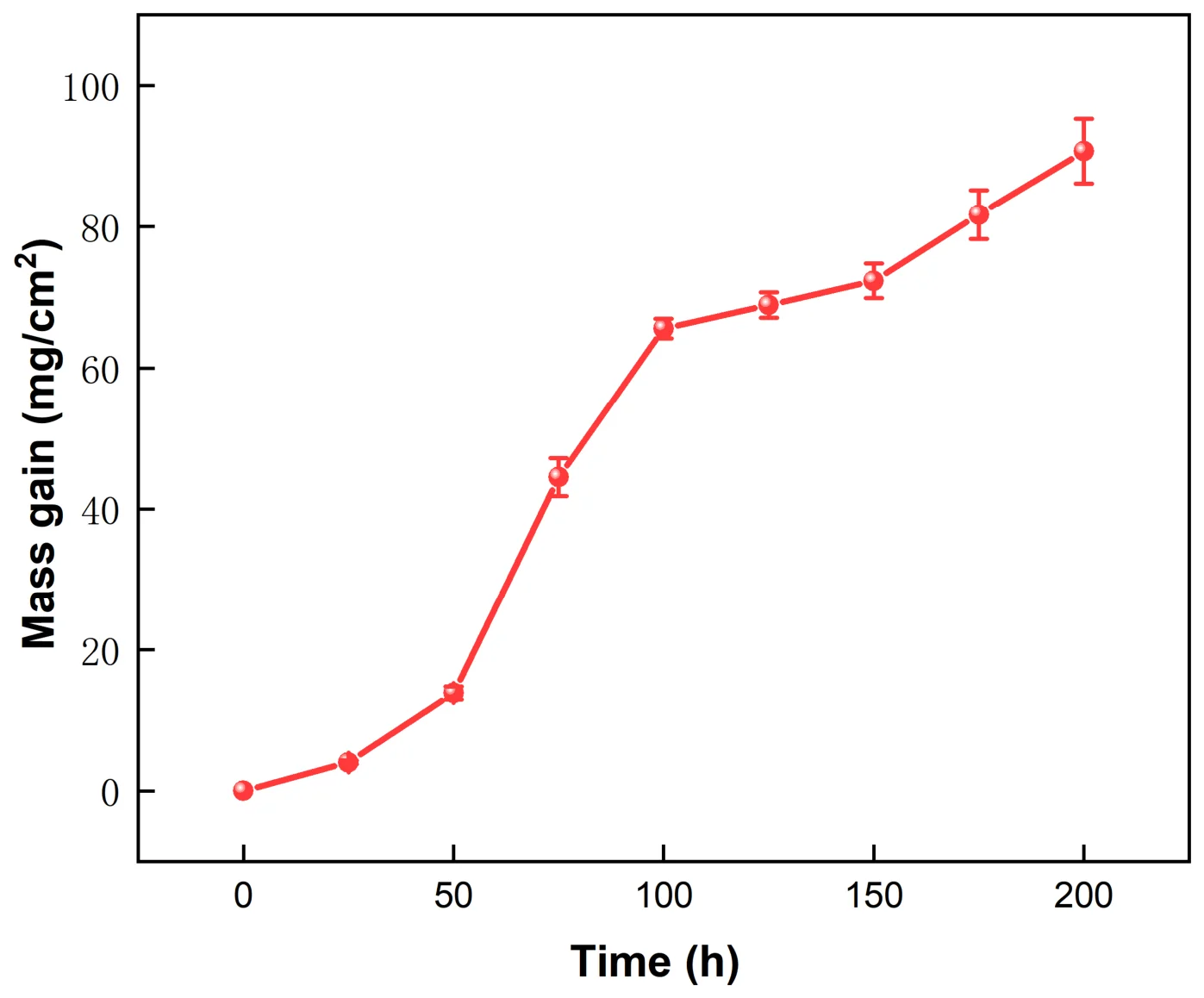
The plot of the weight gain per unit area vs. the exposure time for the SX superalloy specimen at 950 °C.

**Figure 4:**
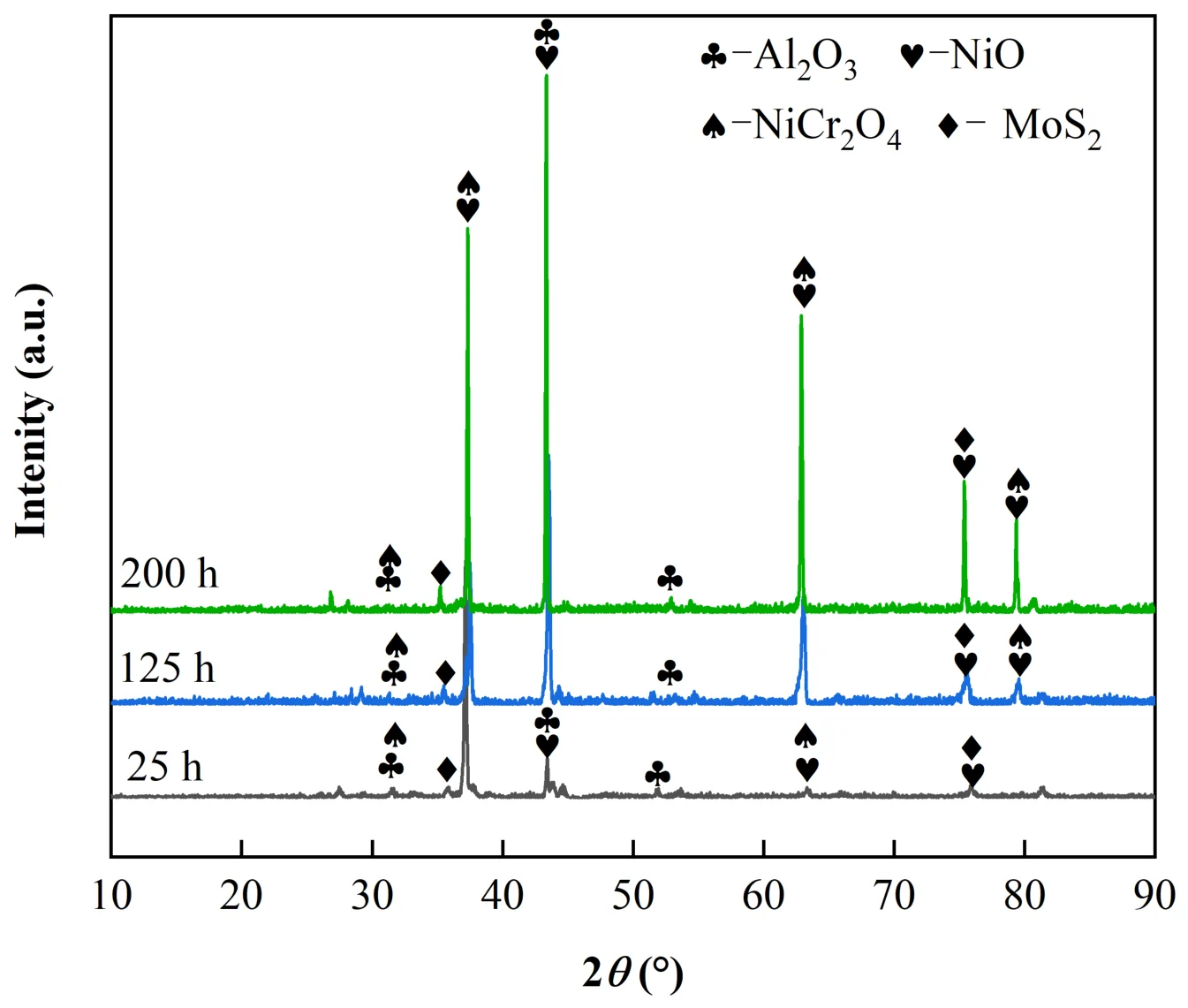
XRD patterns from the corrosion products of the specimens corroded at 950 °C.

**Figure 5:**
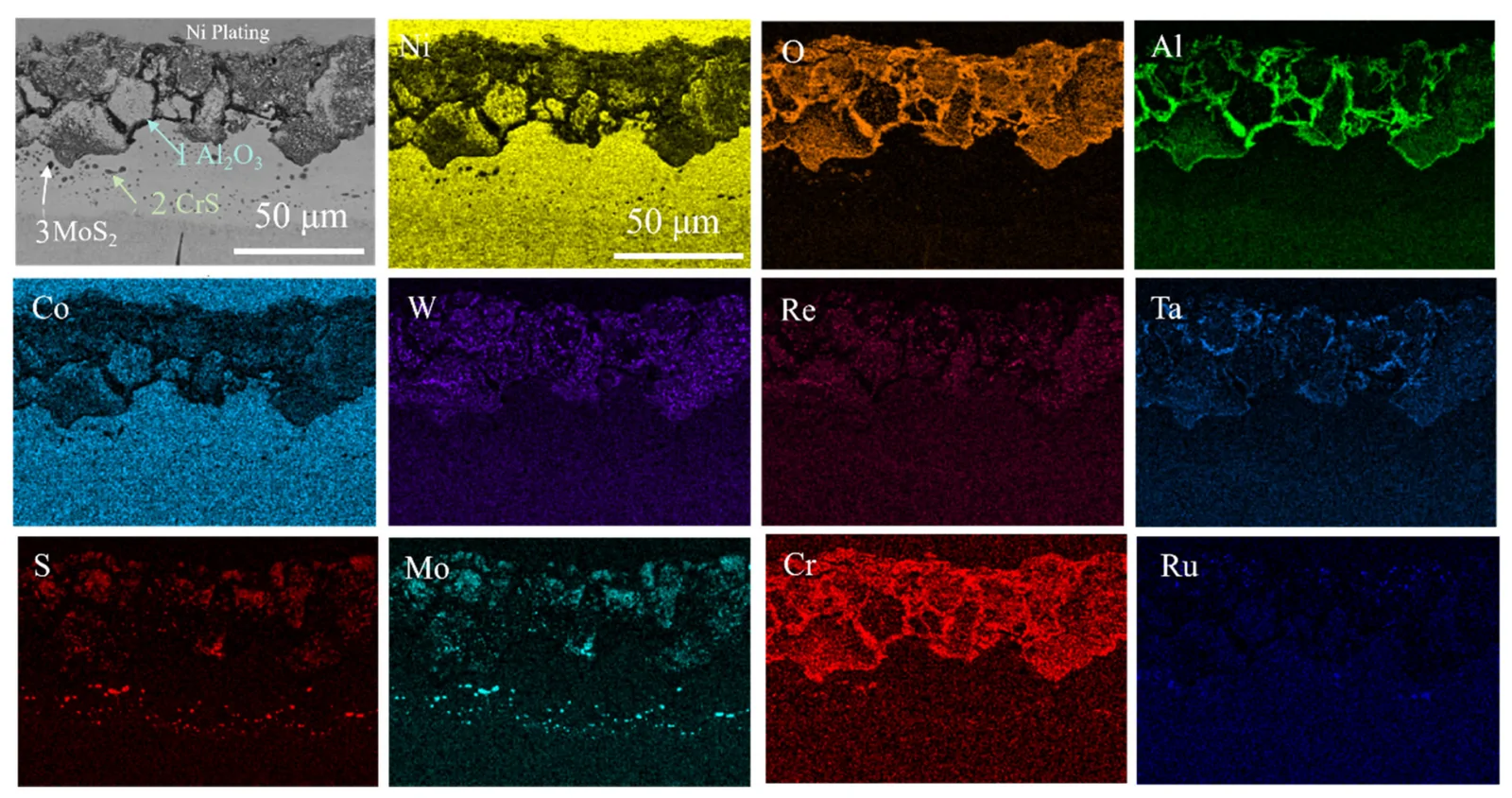
EDX elemental distribution maps of the SX superalloy specimen after exposure in a hot corrosion environment with the Na_2_SO_4_ deposit at 950 °C for 25 h.

**Figure 6:**
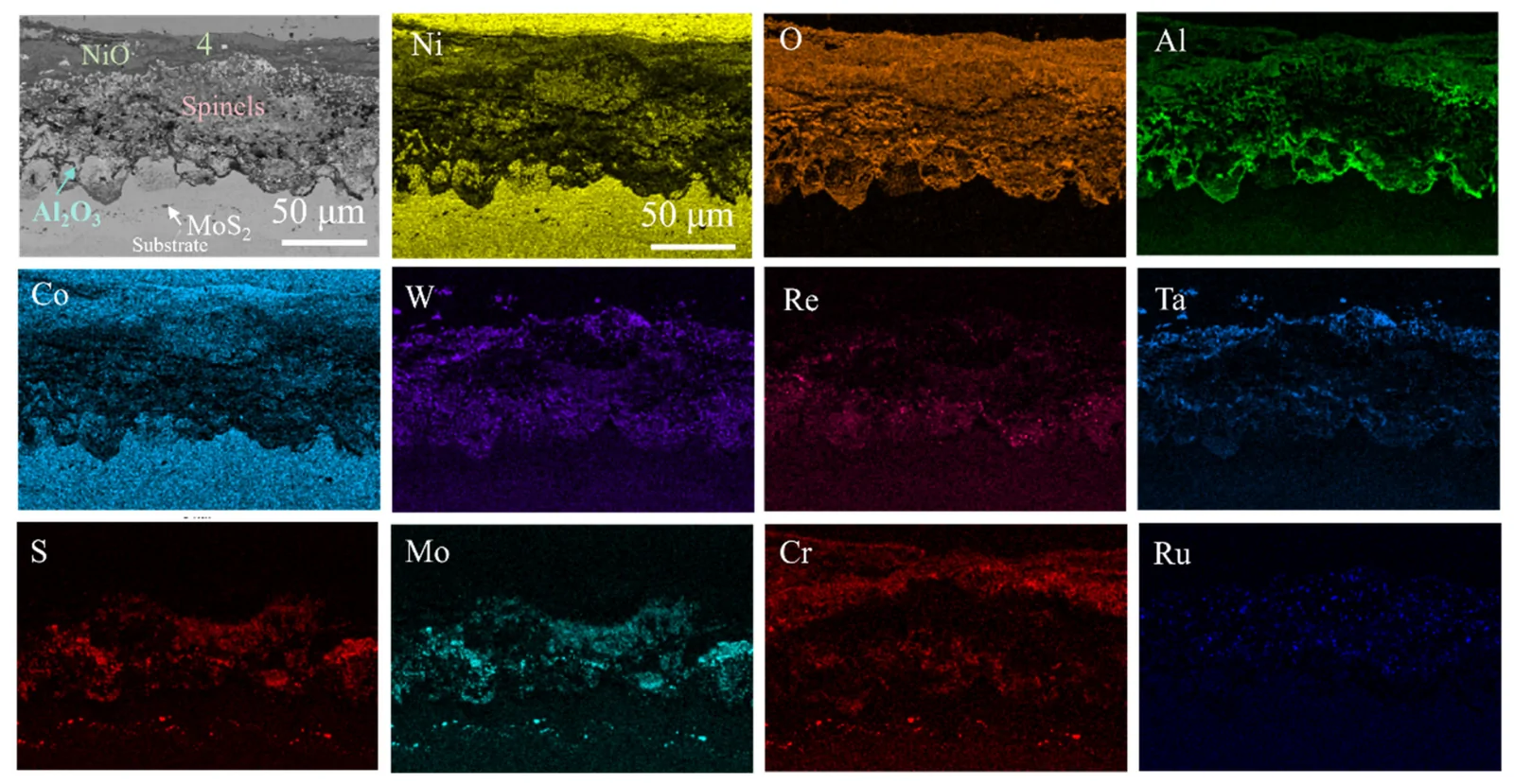
EDX elemental distribution maps of the SX superalloy specimen after exposure in a hot corrosion environment with the Na_2_SO_4_ deposit at 950 °C for 125 h.

**Figure 7:**
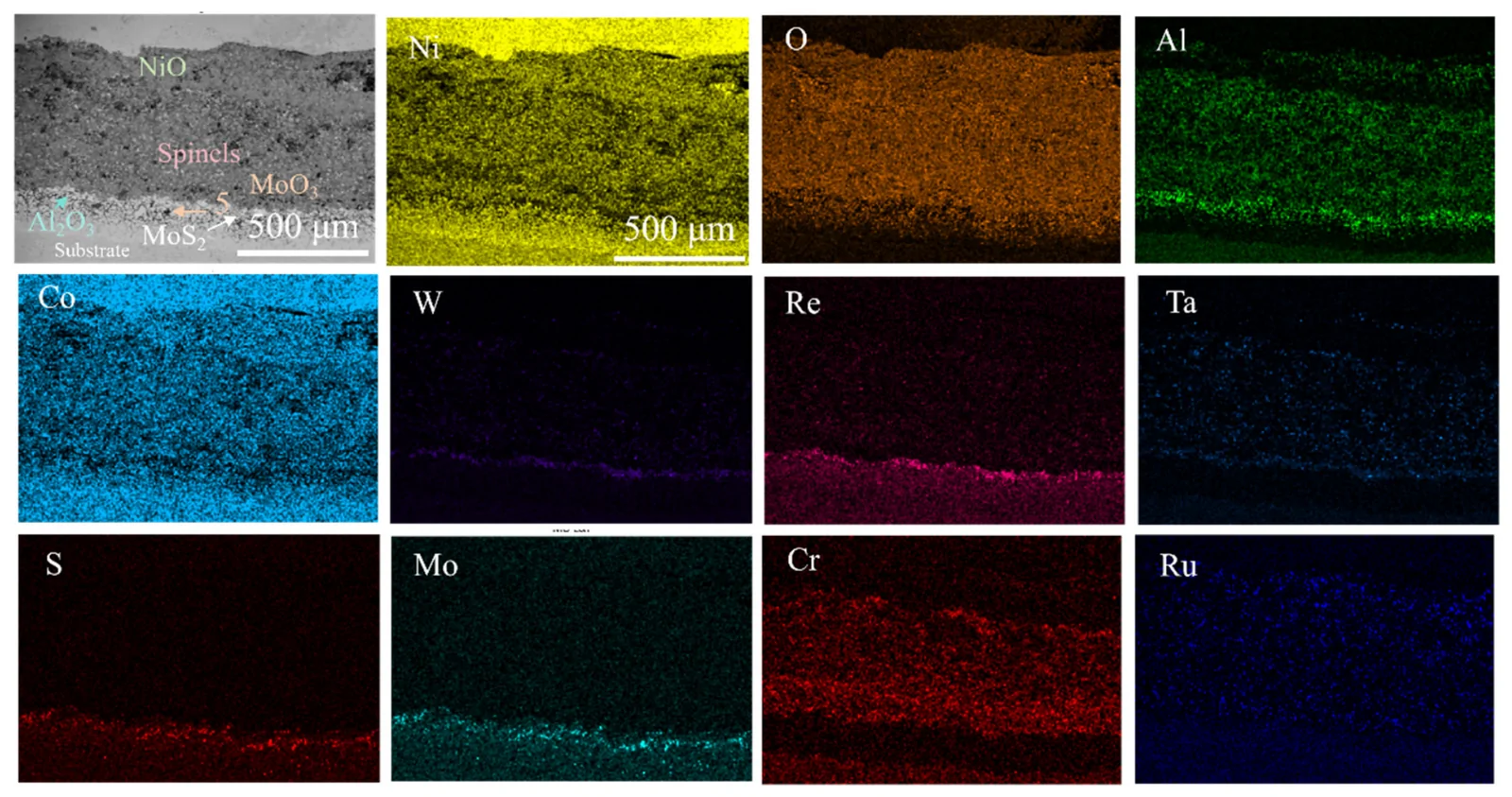
EDX elemental distribution maps of the SX superalloy specimen after exposure in a hot corrosion environment with the Na_2_SO_4_ deposit at 950 °C for 200 h.

**Figure 8:**
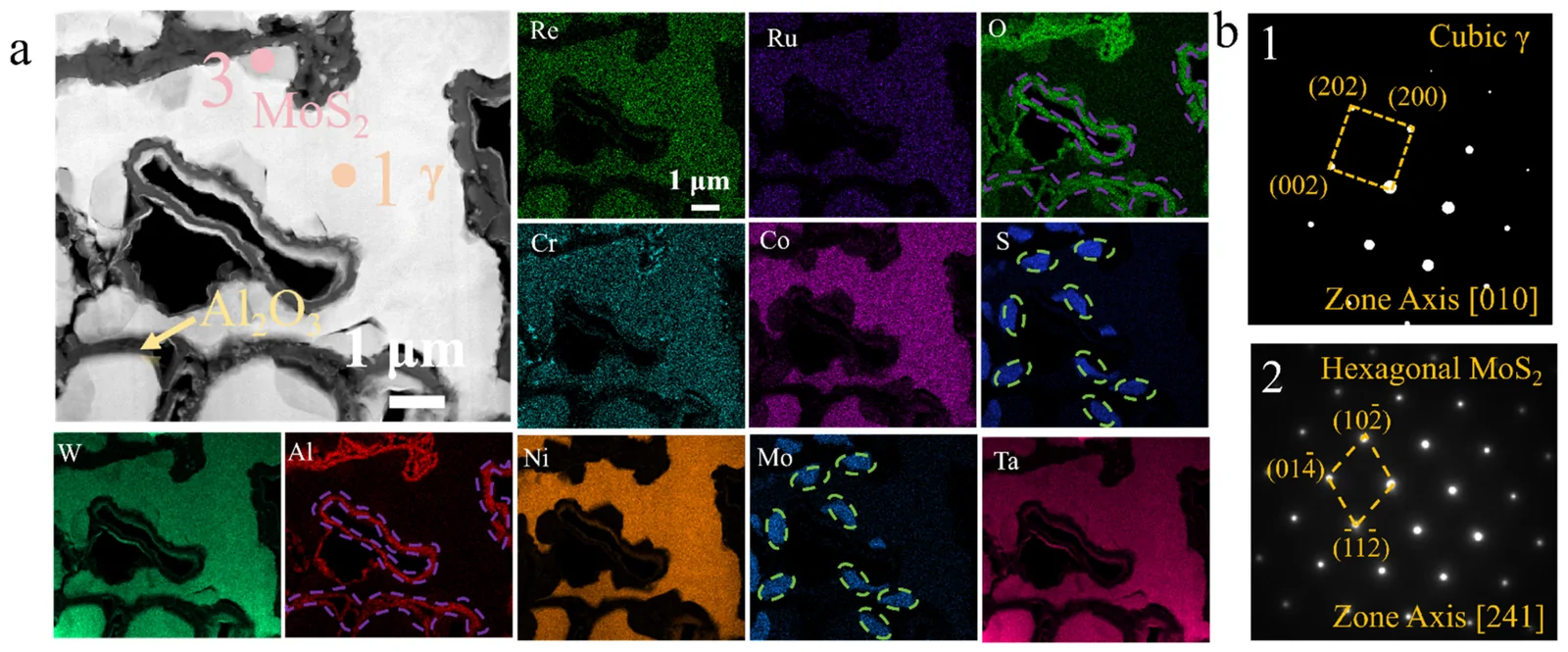
(**a**) The HAADF-STEM image and EDX elemental maps of the oxide layer formed on the specimen surface corroded at 950 °C for 25 h. (**b**) The patterns for Points 1 and 2 are shown in (**a**).

**Figure 9:**
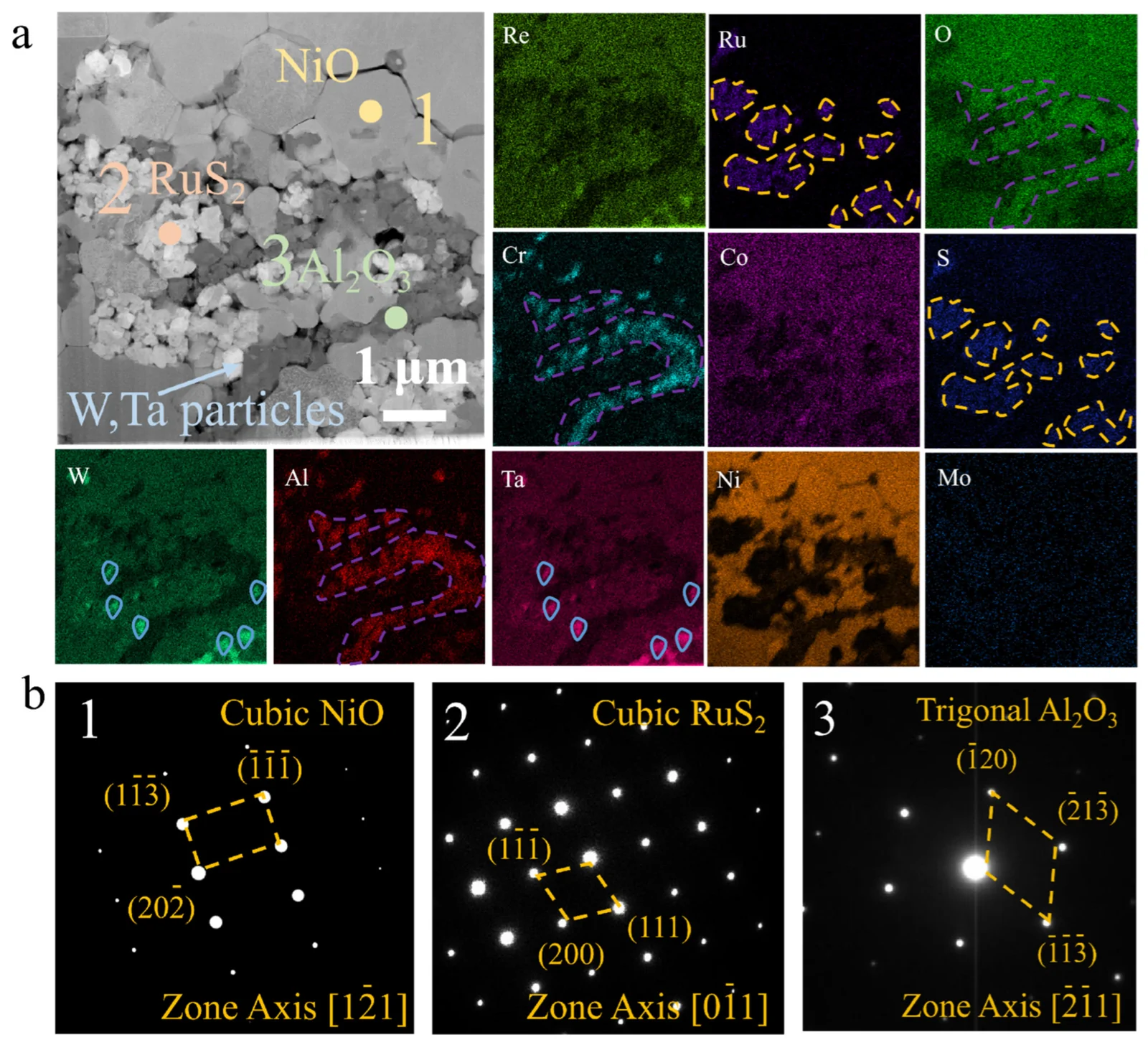
(**a**) The HAADF-STEM image and EDX elemental maps of the oxide layer formed on the specimen surface corroded at 950 °C for 125 h. (**b**) The patterns for Points 1, 2 and 3 are shown in (**a**).

**Figure 10:**
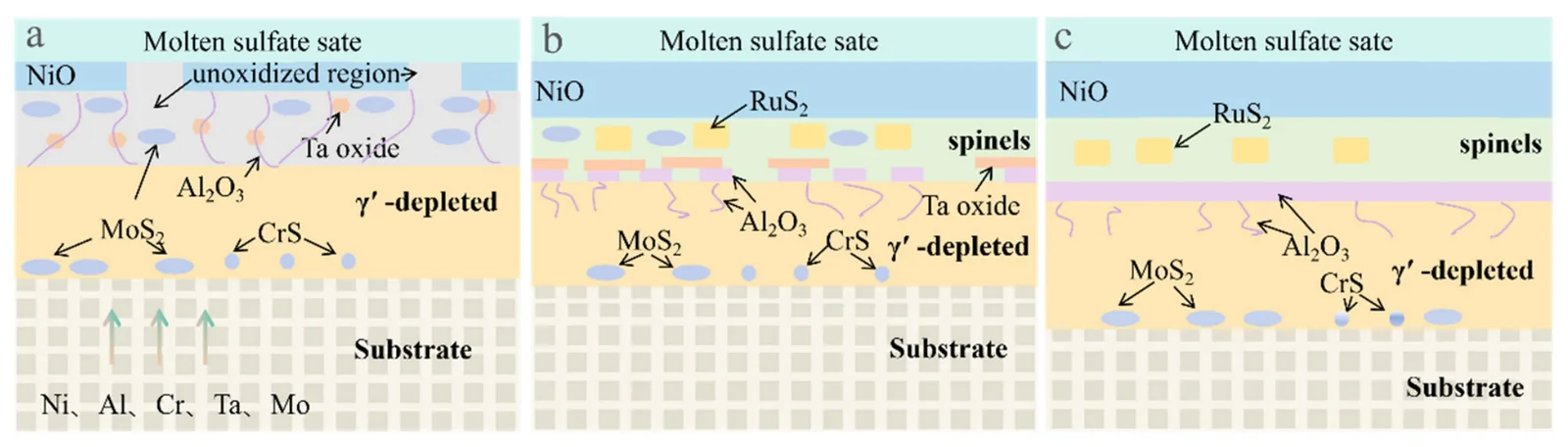
Schematic illustrations of hot corrosion processes of the present SX alloy at 950 °C. (**a**) The incubation stage; (**b**) The early acceleration stage; (**c**) The later acceleration stage.

**Table 1:** Composition of the Nickel-based SX superalloys (wt.%).

	Ni	Al	Co	Cr	Ta	W	Mo	Re	Ru
wt.%	Bal.	5.99	6.04	2.93	4.47	3.97	4.00	5.97	3.76

**Table 2:** Compositions of the five points in Figure 5, Figure 6 and Figure 7 (at.%).

Oxide	Point	O	Ni	Al	Co	Cr	Mo	S
Al_2_O_3_	1	56.3	6.24	34.5	-	0.21	-	2.65
CrS	2	0.4	4.31	0.42	-	40.6	-	50.9
MoS_2_	3	0.3	3.2	0.56	-	-	31.2	59.8
NiO	4	46.3	44.7	-	8.97	-	-	-
MoO_3_	5	66.8	3.08	3.94	-	1.24	24.1	-

**Table 3:** Compositions of the two points in Figure 8 (at.%).

Oxide	Point	O	Ni	Al	Re	Cr	Mo	Ru	S
γ	1	5.37	76.54	1.60	3.17	0.74	0.36	2.24	0.63
MoS_2_	2	3.59	1.34	1.07	1.78	0.90	33.18	1.89	56.24

## Data Availability

The original contributions presented in this study are included in the article/Supplementary Materials. Further inquiries can be directed to the corresponding author.

## Supplementary Materials

The following supporting information can be downloaded at: https://www.mdpi.com/article/10.3390/nano16191225/s1, Figure S1: Selected-area electron diffraction (SAED) pattern of RuS_2_.
