# Supplementary material for: Hot Corrosion Behavior and Mechanism of Ru-Containing Ni-Based Single-Crystal Superalloy Exposed to Molten Na2SO4 at 950 °C
Source: Nanomaterials (Basel). 2026 Sep 28;16(19):1225. doi: 10.3390/nano16191225 (PMC13666578; doi:10.3390/nano16191225)
Supplement: Supplementary file 1 [file nanomaterials-16-01225-s001.zip › nanomaterials-4571268-supplementary.pdf]

Supplementary Materials for

Hot Corrosion Behavior and Mechanism of Ru-Containing Ni-Based Single-Crystal Superalloy Exposed to Molten Na_2SO_4 at 950 °C

Shijia Guo [1,2], Zhihan Wu [1], Shengyun Yuan [1], Bohou Zhang [1], Cheng Zhou [2], Fangmiao Duan [3], Guijuan Zhou [3], Zhigang Li [4], Lianxu Yu [4], Yangtao Zhou [2], Baobing Zhang [5,*] and Yong Zhang [1,*]

[1] Herbert Gleiter Institute of Nanoscience, School of Materials Science and Engineering, Nanjing University of Science and Technology, Nanjing 210094, China
[2] Liaoning Academy of Materials, Shenyang 110167, China
[3] China United Gas Turbine Technology Co., Ltd., Beijing 100016, China
[4] Metalink Special Alloys Corporation, Nanjing 211135, China
[5] Shenyang National Laboratory for Materials Science, Institute of Metal Research, Chinese Academy of Sciences, Shenyang 110016, China
* Correspondence: bbzhang@imr.ac.cn (B.Z.); yong@njust.edu.cn (Y.Z.)

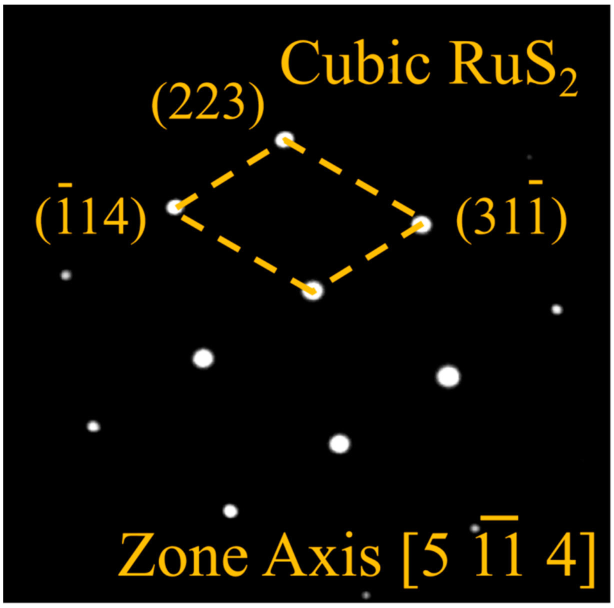

Figure S1. Selected-area electron diffraction (SAED) pattern of RuS_2.
